# Drug Development Inspired by Natural Products II

**DOI:** 10.3390/molecules28217243

**Published:** 2023-10-24

**Authors:** Patricia Rijo

**Affiliations:** 1 Research Center for Biosciences & Health Technologies (CBIOS), 1749-024 Lisboa, Portugal; patricia.rijo@ulusofona.pt; 2 iMed.ULisboa—Research Institute for Medicines, 1649-003 Lisboa, Portugal

For centuries, natural products have been rich sources of healing compounds for different cultures. Today, they continue to hold significance in traditional and alternative medicine. These natural substances exhibit remarkable diversity and potency, solidifying their pivotal role in modern drug development. Recently, there has been rising interest in enhancing these natural compounds through chemical modification and computer-based research. This allows us to maximize their effectiveness as medicines. In this second volume of our Special Issue, we aim to share the latest advancements in drug development inspired by nature.

This Special Issue brings together three valuable contributions, namely a review, an original article, and a communication, all focused on the theme of “Drug Development Inspired by Natural Products”. A review article by Ourdia-Nouara Kernou et al. examines the antimicrobial properties of rosmarinic acid and its derivatives, highlighting their potential as natural antimicrobial agents and synergistic partners with antibiotics in combatting a range of microbial pathogens while addressing mechanisms of action.

The study by Neveen M. Ellboudy et al. investigates the potential antibacterial and antibiofilm properties of cinnamon oil extract, which exhibits a synergistic effect with colistin against *Staphylococcus aureus*, further enhancing its stability and release profile when encapsulated in liposomes, offering a promising natural and safe option for combatting bacterial infections.

The communication by Monika Owczarek et al. explores the potential of chitosan nanoparticles as carriers of Ginkgo biloba extract (GBE), demonstrating the successful encapsulation and release of GBE and conducting preliminary in vitro tests that reveal cytotoxicity against tumor cells while sparing physiological cell lines, showcasing the promising role of these nanoparticles in delivering active substances for use in medical applications.

Overall, the articles presented in this Special Issue provide insightful perspectives and promising avenues for drug development utilizing natural products, showcasing the potential of rosmarinic acid and its derivatives, cinnamon oil extract, and chitosan nanoparticles as innovative agents in the fight against microbial pathogens and as therapeutic agents for medical applications.

